# Human keratinocyte-derived extracellular vesicles activate the MAPKinase pathway and promote cell migration and proliferation in vitro

**DOI:** 10.1186/s41232-021-00154-x

**Published:** 2021-02-02

**Authors:** Azela Glady, Arno Vandebroek, Masato Yasui

**Affiliations:** 1 Department of Pharmacology, Keio University School of Medicine, 35 Shinanomachi, Shinjuku, Tokyo 160-8582 Japan; 2 Keio University Global Research Institute, Center for Water Biology and Medicine, Keio University, Tokyo, Japan

**Keywords:** Keratinocyte, Fibroblast, MAPKinase, Extracellular vesicle, Cell migration, Wound healing, P38, ERK

## Abstract

**Background:**

Wound healing is a complex biological process and complete skin regeneration is still a critical challenge. Extracellular vesicles (EVs) play essential roles in cell communication and cell regeneration, and recent studies have suggested that EVs may contribute to wound healing, though the molecular mechanisms behind this contribution remain unclear. For these reasons, we decided to use EVs isolated from human keratinocytes (HaCaT) in vitro to determine the potential mechanism of action of EV-derived wound healing.

**Method:**

Scratch assays were used to determine cell migration and proliferation. Scratched cells were exposed to EVs in multiple conditions to determine how they affect wound healing. Statistical analysis between groups was carried out to using Student’s two-sided *t* test. A *p* value of <  0.05 was considered statistically significant.

**Result:**

We found that proteomic analysis of purified EVs shows enrichment of proteins associated with cell communication and signal transduction, such as MAPK pathways, and keratinocyte and fibroblast cultures exposed to EVs had higher levels of proliferation, migration, and ERK1/2 and P38 activation. Moreover, we found that treatment with specific ERK1/2 and P38 signaling inhibitors PD98059 and SB239063 impaired EV-mediated cell migration, which suggests that ERK1/2 and P38 signaling is essential for EV-induced wound healing.

**Conclusion:**

HaCaT cell-derived EVs accelerate the migration and proliferation of human keratinocytes and fibroblasts and may promote wound healing via the activation of MAPKinase pathways. These findings may be key in developing new methods to treat wounds and accelerate wound healing in the future.

**Supplementary Information:**

The online version contains supplementary material available at 10.1186/s41232-021-00154-x.

## Background

Skin is the largest organ in the human body and functions as an important barrier against the external environment by protecting the body from infection and injuries [1–3]. A wound originating from any trauma can damage this barrier, increasing the possibility of infection; therefore, any wound to the skin must be promptly repaired to avoid further complication and chronic infection. Wound healing, especially cutaneous wound healing, is a complex and overlapping biological process which involves a series of steps including inflammation, proliferation, and remodeling [4, 5]. In current medical treatment, wound care has progressed much, from ointment and external dressing which are used to prevent the entry of bacteria into a wound, to grafting, which are used to accelerate wound healing [4]. However, skin grafting is reliant on a supply of a sufficient amount of donor skin, which may frequently be scarred or discolored [6, 7]. Hence, it is still necessary to find a new stable, efficient, and safe method to promote soft tissue wound healing [8, 9].

At present, it is believed that extracellular vesicle (EVs) can become a novel method to accelerate wound healing [10]. EVs are lipid bilayer particles containing RNA and proteins, with a diameter ranging from 30 to 200 nm, that are naturally released from various types of cells during the process of cell communication [11]. In general, EVs contain cytokines, growth factors, RNA, and DNA which are transferable to the target cells [12]. There has been rapid growth in EV related studies for the past several years in various kinds of research areas, particularly the therapeutic area. Zhao et al. showed that treatment with mesenchymal stem cell (MSC)-derived exosomes effectively enhances cutaneous wound healing in mice by activating the human keratinocytes (HaCaT) cells in the skin [13]. Wang et al. also proved that fetal dermal mesenchymal stem cell-derived exosomes could accelerate cutaneous wound healing in vivo [14]. Previous studies have identified an upregulation of VEGF and TGF-α and subsequent migration of fibroblasts to the wound site as a possible mechanism for EV-induced would healing [15, 16], however, it is likely that there are several different pathways through which EVs have an effect on would healing. The purpose of this study is, therefore, to determine whether an alternate pathway exists and, if so, to identify it.

Several studies have pointed out a key role of mitogen-activated protein kinase (MAPK) signaling in wound injury by triggering growth factors and cytokines which stimulate migration to, and proliferation in, the wound edge [17]. Ren et al. mentioned that the activation of extracellular signal-regulated kinase (ERK) signaling promotes cell proliferation and migration in wound healing when using human adipose stem cell vesicles in a mouse wound model [11, 18]. Srinivasan et al. showed that ERK promoted cell proliferation and migration during mouse embryonic angiogenesis [19]. These findings indicate the importance of MAPKinase pathway in cell proliferation and migration.

HaCaT cells are immortalized human keratinocytes which have been used to study various kinds of dermatological conditions such as contact dermatitis, psoriasis, or skin cancer [20–22]. Due to their high availability and ease of cell culture [23], HaCaT cells offer an easily scalable method of acquiring EVs. In addition, the effect of human keratinocyte-derived EVs has not yet been investigated in the context of wound healing. This study, therefore, intends to focus on the basic mechanism through which EVs released from human keratinocytes could improve cell proliferation and migration in vitro and identify the molecular signaling behind that process.

## Methods

### Materials

PD98059 was purchased from Calbiochem (CAS 167869-21-8 #cat 513000). SB239063 was purchased from Calbiochem CA (CAS 798558-40-4 #cat 559404).

### Cell lines

Human keratinocytes (HaCaT) were purchased from Cell Lines Service and cultured in Dulbecco’s modified Eagle’s medium (*DMEM, WAKO*) containing 10% fetal bovine serum (FBS) and 1% penicillin-streptomycin (5% CO_2_, 37 °C) until 80% confluence.

Adult Normal Human dermal fibroblasts (NHDF) were purchased from a commercial supplier (Takara-bio) and were cultured in Dulbecco’s modified Eagle’s medium (*DMEM, WAKO*) containing 10% FBS and 1% penicillin-streptomycin (5% CO_2_, 37 °C) until 80% confluence.

### TIM-4 affinity purification of EVs

Prior to EV isolation, cells were sub-cultured to 20, 10 cm dishes for a total culture volume of 200 ml. Cells were incubated in serum-free media for 24 h before isolation. TIM-4, a phospholipid phosphatidylserine present on the surface of the extracellular vesicle, can be used to isolate EVs from the serum-free media. To isolate EVs by the TIM-4 affinity method, a MagCapture Exosome Isolation Kit PS (Wako, Japan) was used according to the manufacturer’s instructions. In brief, 0.6 mg of streptavidin magnetic beads, bound with 1 μg of biotinylated mouse Tim4-Fc, was added to 10K filtered supernatant and the mixture was rotated overnight at 4 °C. The beads were washed three times with 1 ml of washing buffer (20 mM Tris-HCl, pH 7.4, 150 mM NaCl, 0.0005% Tween20, 2 mM CaCl_2_), and the bound EVs were eluted with elution buffer (20 mM Tris-HCl, pH 7.4, 150 mM NaCl, 2 mM EDTA).

### Cell lysates

Cell lysates were acquired from HaCaT cells. Cell suspensions (of approx. 4 × 10^6^ cells) were centrifuged at 300×*g* for 5 min, the supernatant discarded, and the pellet resuspended in PBS followed by a second centrifugation step. The pellet was then resuspended in radioimmunoprecipitation assay buffer (RIPA, Sigma) with 1% protease/phosphatase inhibitor cocktail (Cell Signaling Technology) and resuspended via pipetting for 5 min. Finally, the samples were centrifuged at 8000×*g* for 10 min at 4 °C and the supernatant was collected and aliquoted for downstream analyses.

### Total protein concentration measurement

Total protein concentration was measured using a Pierce™ MicroBCA Protein Assay Kit (ThermoFisher Scientific). The exosomes were diluted 1:1 with RIPA, pipetted for 5 min, diluted in MilliQ water (Merck Millipore) and a BCA assay was subsequently performed according to manufacturer’s instructions.

### Western blotting

Cell lysates or EVs were diluted 1:1 in LDS Sample Buffer (Thermo Fisher Scientific) and heated to 95 °C for 5 min and subsequently cooled on ice prior to immunoblotting with antibodies. Blocking, antibody incubation, and washing steps were performed on rocking platforms. Blocking was done using 5% dry milk (Cell Signaling Technologies) in TBS-T for 1 h and the membranes were exposed to primary antibody in 4 °C overnight. Membranes were then washed 3 × 10 min in TBS-T on an orbital shaker and exposed to secondary antibody for 60 min in room temperature. The membranes were washed 3 × 10 min using TBS-T, and the buffer was drained from the filter and detection reagent (ECL Prime WB detection Kit, Sigma-Aldrich) was added for 5 min. The signals were then detected using LAS 4000 mini (Fujifilm).

The following antibodies and concentrations were used: CD9 (#13174, Cell Signaling Technology, 1:1000), CD63 (#10628D, Thermo Fisher Technologies, 1:1000), phosphor-ERK (#3191, Cell Signaling Technology, 1:1000), ERK (#3192, Cell Signaling Technology, 1:1000), phosphor-p38 (#9211, Cell Signaling Technology, 1:1000), p38 (#9212, Cell Signaling Technology, 1:1000), and Anti-rabbit IgG, HRP-linked (#7074, Cell Signaling Technology, 1:2000).

### Mass spectrometry and bioinformatic analysis

The original MS/MS file data from Cosmo Bio Co. Ltd. was imported into FunRich Software 3.1.3 for data analysis. The sequences of the identified proteins were mapped against the Gene Ontology (GO) database to determine their biological and functional properties. Three main types of annotations, cellular components, molecular functions, and biological process, were obtained from the GO consortium website.

### Scratch assay

For wound healing and cell migration assays, HaCaT and Fibroblast cells were seeded in 24-well plates at 0.5 × 10^6^ cells/ml. Twenty-four hours later, the medium was changed to non-FBS-containing media. The following day confluent cells were uniformly scraped with a 200-μl pipette tip across the well. Following wounding, culture medium was washed with PBS and replaced with fresh serum-free medium, and cells were continually exposed to EVs for the indicated time periods. The scratched region was photographed immediately and every 12 h after scratching using an EVOS M5000 microscope (Invitrogen) at × 20 magnification. Total scratch area was analyzed using ImageJ.

### Statistical analysis

GraphPad Prism v8.0 (GraphPad Software) was used for statistical analyses. Statistical analysis between two groups was carried out to using Student’s two-sided *t* test. A *p* value of < 0.05 was considered statistically significant. Significance levels and exact *p* values were indicated in all relevant figures. Data were assumed to be normally distributed for all analyses conducted. Data for independent experiments were presented as means ± SEM (standard error of the mean) unless otherwise stated.

## Results

### Characterization of human keratinocyte-derived extracellular vesicles

**Fig. 1:**
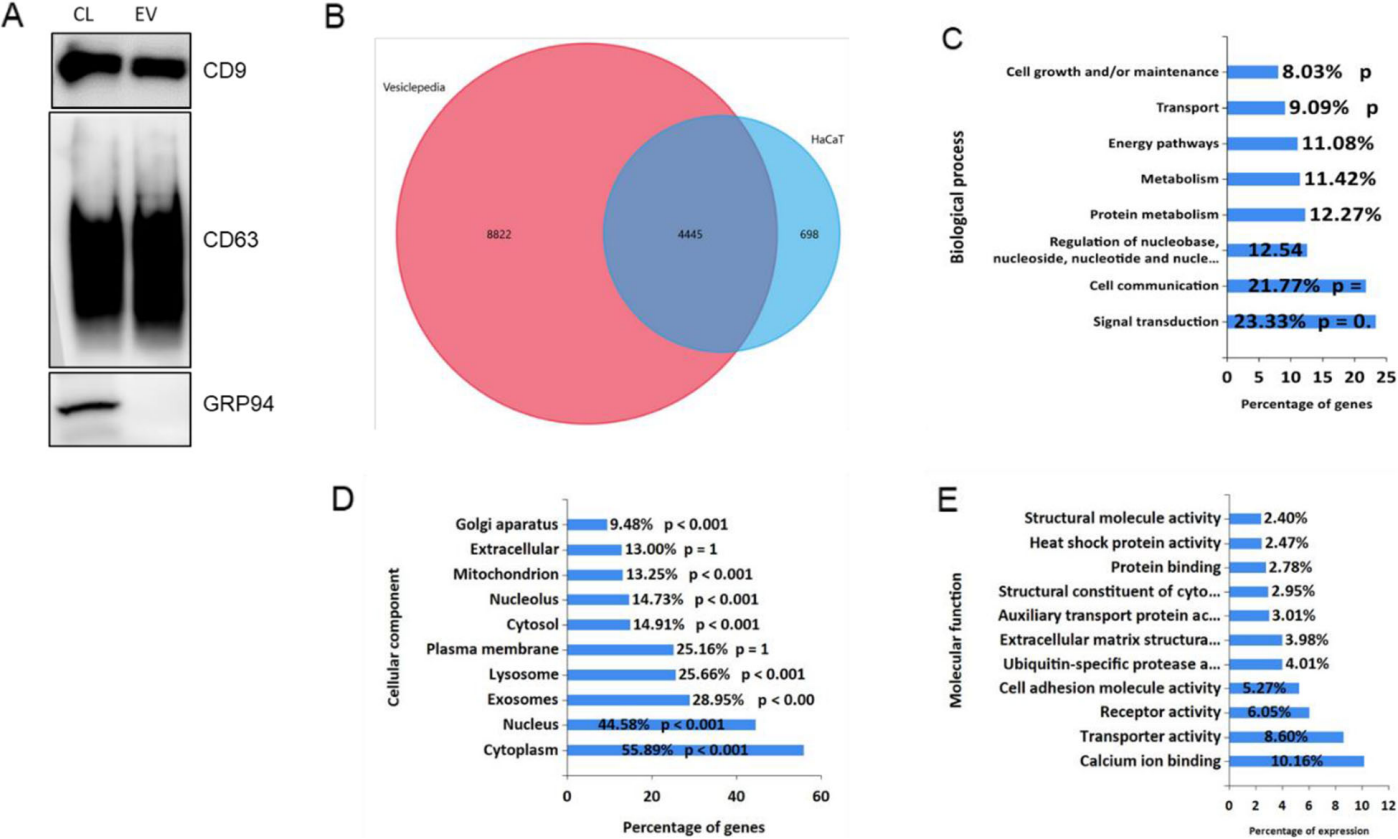
Characterization of extracellular vesicles (EVs) released by HaCaT cells. **a** Western blotting analysis of purified vesicles (EV) for exosomal surface markers (CD9, CD63) and endoplasmic reticulum marker (GRP94) side by side with HaCaT cell lysate (CL). **b** Venn diagram of identified proteins and proteins in the vesiclepedia database. The pink area represents proteins from the vesiclepedia database and the blue area represents proteins we identified. The identified proteins in HaCaT cell-derived EVs were analyzed using FunRich Bioinformatic Resources and classified by **c** biological process, **d** cellular component, or **e** molecular function

In this study, we used human keratinocyte (HaCaT) cells to investigate the effect of EVs on cellular proliferation, migration, and associated mechanisms. EVs from HaCaT cell condition media were isolated by TIM-4 affinity purification and characterized via Western blot. CD9 and CD63 were used as markers for EVs, while GRP94, a protein found in the endoplasmic reticulum of HaCaT cells, was used to assess purity of the EV sample. In Fig. 1a, the blots of CD9 and CD63 proteins reveal distinct traces, indicating a positive EV signal, while GRP94 is only found in the cell lysate (CL), and not the purified EV fraction, suggesting that the EV purification method was successful and the EV fraction is free of contaminating proteins.

To examine the proteomic profile of EVs secreted by HaCaT cells, liquid chromatography with tandem mass spectrometry (LC-MS/MS) analysis was performed. The resulting MS/MS spectra were analyzed using data-independent acquisition (DIA) and searched against Vesiclepedia, a database containing proteins which have been identified in EVs [24]. The comparison against Vesiclepedia showed the identification of 698 unique proteins (Fig. 1b, STable 1). As shown in the Venn diagram, a total of 4445 proteins were common to Vesiclepedia and 698 proteins are specific to HaCaT EVs.

By comparing against the Gene Ontology (GO) database [25, 26] in FunRich Bioinformatic Resources, EV-derived proteins were categorized by biological process (Fig. 1c), cellular component (Fig. 1d), and molecular function (Fig. 1e). The biological processes associated with the identified proteins were focused on signal transduction (23.33%) and cell communication (21.77%). Of the major cellular components, the cytoplasm, nucleus, and exosomes were the most highly represented classifications. Certain functional activities such as calcium ion binding, transporter activity, and receptor activity were represented, suggesting that MAPKinase-related cell communication and signal transduction may be essential for the biological function of HaCaT cell-derived EVs (Fig. 1e).

### EVs promote cell migration in vitro

**Fig. 2:**
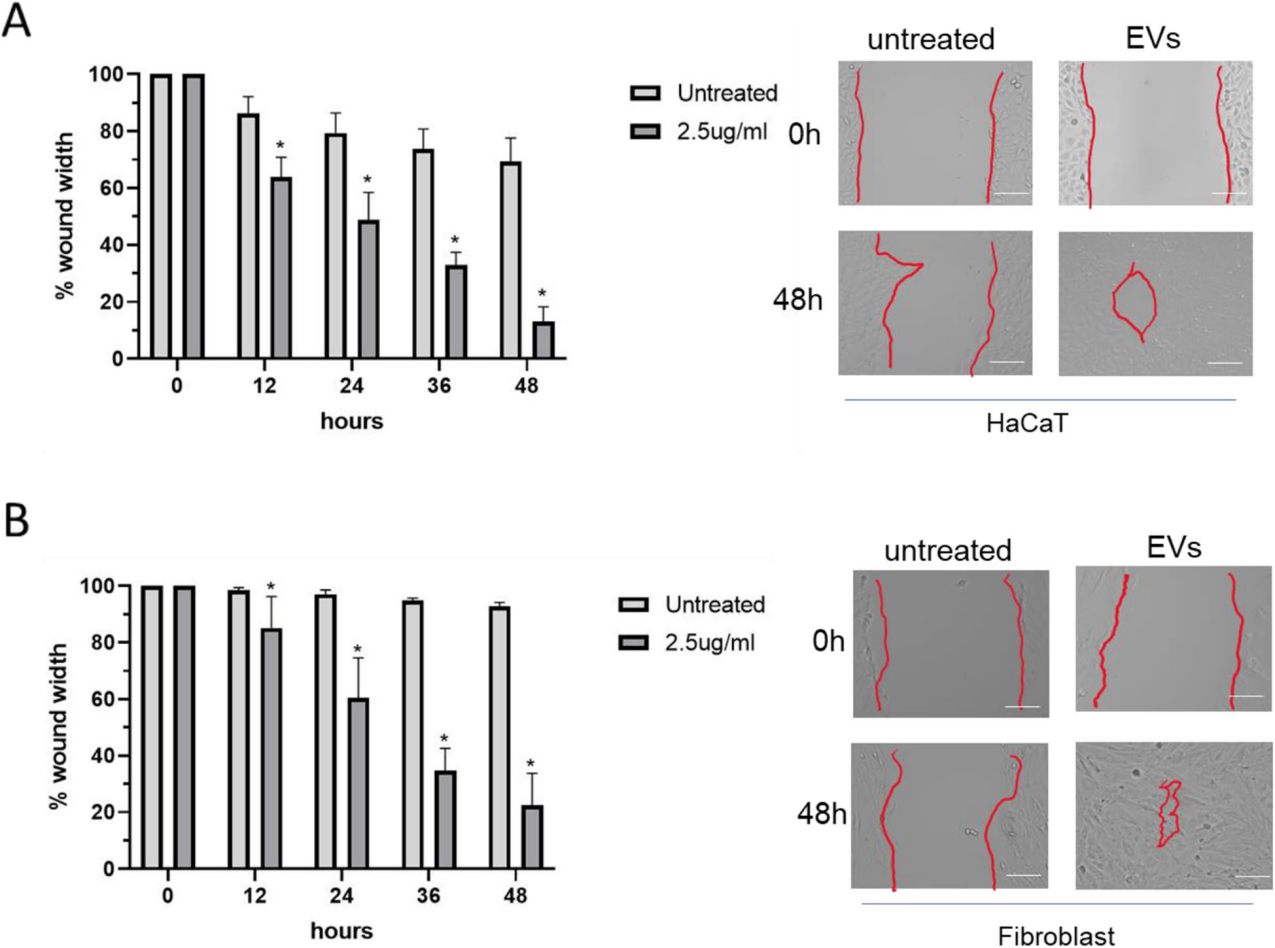
HaCaT-derived EVs promote cell migration in vitro. **a** HaCaT cell scratch assay results after exposure to 2.5 μg/ml HaCat cell-derived EVs or left untreated for 48 h. **b** Fibroblast cell scratch assay results after exposure to 2.5 μg/ml HaCaT cell-derived EVs or left untreated for 48 h (*n* = 24 scratches). Red lines indicate scratch boundaries. Scale bar = 100 μm

Extracellular vesicle have long been known to be a form of cell communication [27]. H. Paul Ehrlich mentioned that cell communication is important for wound healing, and these communication events are concentrated along the wound edge and are reduced in cells further away from the wound [28]. With that in mind, a greater understanding of cell to cell interaction and communication will provide evidence for the role of cell communication in influencing cell migration and proliferation. The proteomic analysis results show that 21.77% of the identified proteins were classified into the category of cell communication (Fig. 1b). This may be the reason that extracellular vesicles may trigger cell proliferation and migration. The ability of HaCaT EVs to stimulate HaCaT cell proliferation and/or migration was visualized by using a scratch assay. After optimization of EVs concentration (SFig. 1), treatment with 2.5 μg/ml HaCaT-derived EVs significantly enhanced the migration of HaCaT cells to the scratch area after 48 h compared to the control group (Fig. 2a), *P* = 0.002 in 12 h, *P* = 0.002 in 24 h, *P* = 0.0001 in 36 h, and *P* = 0.00007 in 48 h. We next pondered whether HaCaT-derived EVs could induce the same effect on fibroblast cells. We confirmed that EVs derived from HaCaT cells could promote migration in fibroblast cells as shown in Fig. 2b, *P* = 0.04 in 12 h, *P* = 0.01 in 24 h, *P* = 0.0005 in 24 h, and *P* = 0.0009 in 48 h (asterisk indicates significant different between untreated and treated). Taken together, these results indicate that EVs from HaCaT cells can play a key role in accelerating cell migration in vitro.

### The MAPKinase pathway is activated by EVs

**Fig. 3:**
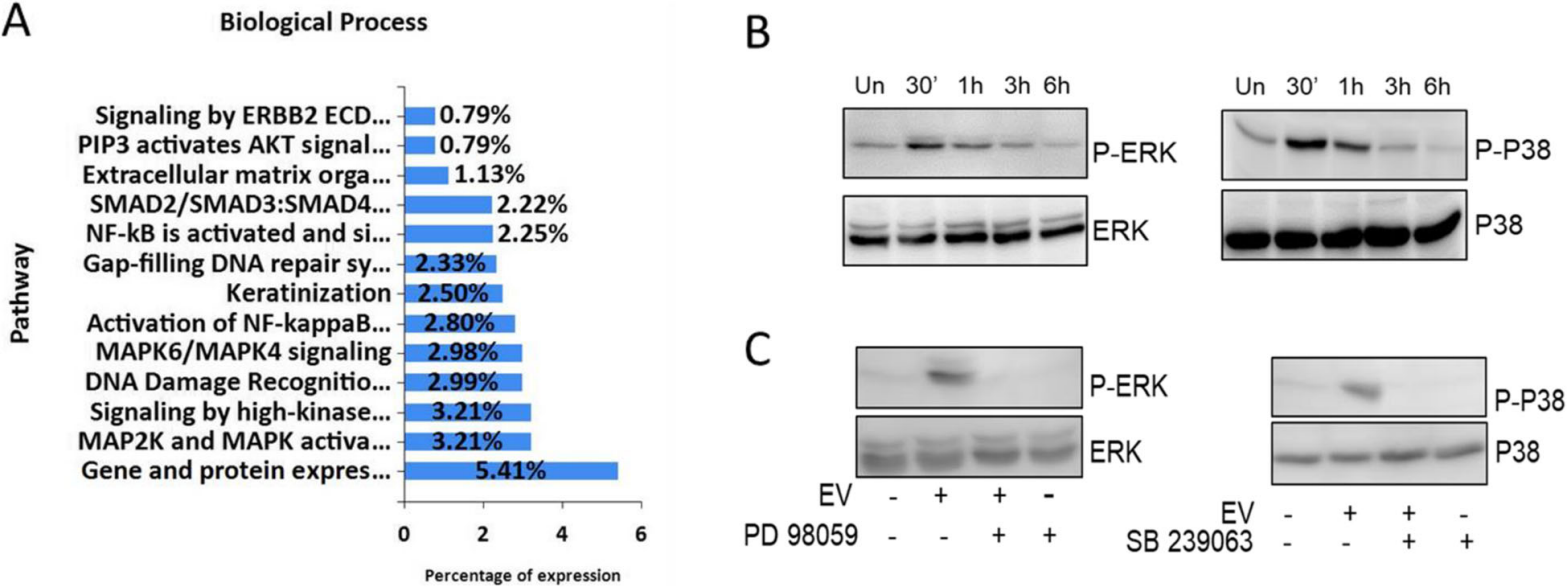
Biological process pathway of HaCaT-derived EVs (**a**). Classification of HaCaT EV proteins by biological process pathway from the proteomic analysis (**b**). Western blotting indicating the expression of P-ERK1/2, ERK1/2, P-P38, and P38 by HaCaT cells after incubation with 2.5 μg/ml EVs for 30 min, 1 h, 3 h, and 6 h. **c** HaCaT cells were exposed to 2.5 μg/ml EVs for 30 min in the absence or presence of 10 μM SB239063 or 10 μM PD98059

EVs have been shown to transfer information between cells [29]. Our proteomic analysis findings show that EV-derived proteins strongly associated with the MAPKinase pathway (Fig. 3a). Therefore, we investigated the phosphorylation status of few key components of the MAPKinase pathway following the introduction of 2.5 μg/ml HaCaT-derived EVs into the culture media of HaCaT cells using western blotting. As seen in Fig. 3b, our findings show that EVs induce a time-dependent phosphorylation of ERK1/2 (P-ERK1/2) and P38 (P-P38). The phosphorylation of ERK1/2 and P38 was at its highest after 30 min of EV exposure and had returned to normal levels by 3 h post exposure. Interestingly, at 6 h post exposure, phosphorylation levels are lower than the untreated group. This could potentially be due to regulation via a negative feedback loop. These results strongly suggest that the activation level of ERK and P38 signaling pathways in HaCaT cells was augmented by HaCaT-derived EVs.

To determine how EVs activate ERK1/2 and P38, cells were treated with a MEK1/2 inhibitor (PD98059), and a P38 inhibitor (SB239063), and then incubated with EVs for 30 min (Fig. 3c). Western blotting was performed to detect the phosphorylation state and expression of the related molecules. Pre-treatment of HaCaT cells with PD98059 or SB239063 inhibited the increased P-ERK and P-P38 induced by the EVs, suggesting that, at least in the case of ERK, the EVs do not activate these proteins themselves but likely activate upstream components of the MAPKinase pathway.

### EVs enhance cell migration via the MAPKinase pathway

**Fig. 4:**
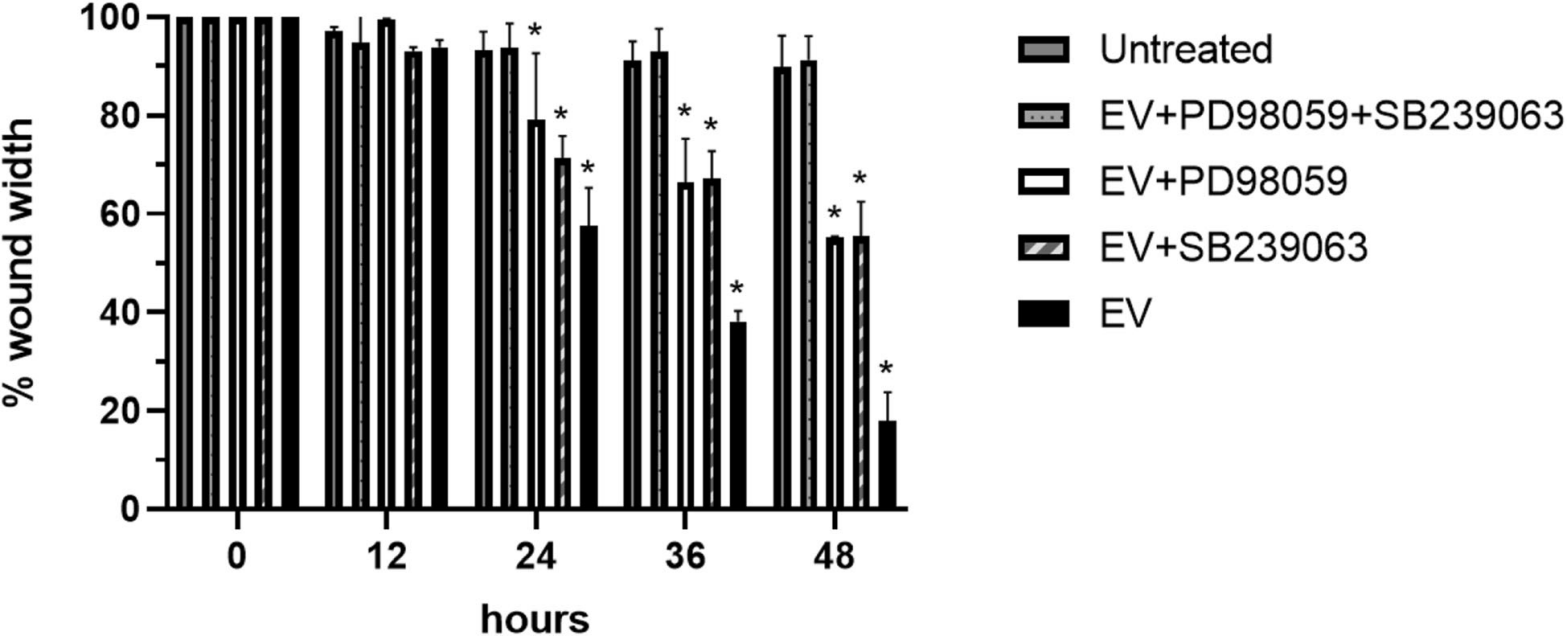
Involvement of the MAPKinase pathway in cell migration in vitro. HaCaT cell scratch assay results after exposure to HaCaT cell-derived EVs in the presence and absence of MEK1/2 and P38 inhibitors for 48 h (*n* = 8 scratches). Asterisk indicates all treatment groups are compared to untreated

To determine whether the activation of the MAPKinease pathway by EVs altered cell migration, we used a scratch assay in which EVs were added to the culture media in the presence or absence of MEK1/2 inhibitor (PD98059) or P38 inhibitor (SB239063). In this model, the migration of HaCaT cells was significantly enhanced by EV treatment for 48 h compared with the untreated control (*P* = 0.02 in 24 h, 0.003 in 36 h, and 0.007 in 48 h). This enhanced migration was partially mitigated when EV-treated cells were exposed to 10 μM PD98059 or SB239063 for 48 h (against untreated, *P* = 0.03 in 24 h, *P* = 0.04 in 36 h, and *P* = 0.01 in 48 h for PD98059) (*P* = 0.03 in 24 h, *P* = 0.03 in 36 h, and *P* = 0.03 in 48 h for SB239063). Moreover, treatment with both inhibitors together completely inhibited the migration of HaCaT cells (*P* = 0.93 in 24 h, *P* = 0.72 in 36 h, and *P* = 0.8 in 36 h). Treatment with both inhibitors was significantly different with single inhibitor (against EV+SB239063, *P* = 0.04 in 24 h, *P* = 0.03 in 36 h, and *P* = 0.02 in 48 h, and against EV+PD98059, *P* = 0.04 in 24 h, *P* = 0.03 in 36 h, and *P* = 0.01 in 48 h), indicating that multiple routes of the MAPKinase pathway are used to promote cell migration. There is no significant difference between each single inhibitor group (*P* = 0.98 in 24 h, *P* = 0.90 in 36 h, and *P* = 0.91 in 48 h), and treatment with EV alone is significantly different from all inhibitor groups (against both inhibitors, *P* = 0.03 in 24 h, *P* = 0.004 in 36 h, and *P* = 0.005 in 48 h; against EV+SB239063, *P* = 0.02 in 36 h and *P* = 0.03 in 48; and against EV+PD98059, *P* = 0.02 in 24 h and *P* = 0.01 in 36 h). Treatment of cells with PD98059 or SB239063 or multiple inhibitors in the absence of EVs had no significant effect on cell migration compared to the untreated control (SFig. 3) (*P* > 0.05). These results indicate that EV treatment likely affects multiple upstream components of the MAPKinase pathways, as treatment with only a single inhibitor still shows a significant effect on cell migration (Fig. 4, SFig. 2).

## Discussion

Skin is the largest organ of the human body and has many functions; therefore, rapid healing of skin wound is necessary [30]. As regeneration processes take place in wound healing, keratinocyte migration determines the efficiency of the initial wound healing process [31]. In this study, we have shown that EVs derived from human keratinocytes have a remarkable effect on cell migration via the MAPKinase pathway.

Extracellular vesicles are lipid bilayer compartment containing lipids, protein, and nucleotides [32]. EVs are present in all biological fluids released by all types of cells in human body and function as a method of cell communication [32]. Previous research recently showed that EVs are associated with wound healing processes in vivo and in vitro [15]. Exosomes derived from human umbilical cord mesenchymal stem cells (MSC) enhance proliferation and migration of skin cells via Wnt4-mediated β-catenin nuclear translocation [33, 34]. Zhao et al. used human amniotic derived exosome to accelerate diabetic mice’s wound healing [35]. However, the molecular mechanism and cell signaling within EVs that mediated wound healing is still unclear. We are eager to explore the molecular and cell signaling using in vitro methods. To our knowledge, this is the first study to investigate treatment of HaCaT cells with their own EVs in the context of wound healing. In our work, we have successfully isolated EVs from HaCaT cells. Proteomic analysis was performed, and 5143 proteins were identified through LC-MS/MS. The gene symbols of identified EV proteins were compared with those in Vesiclepedia, a gene symbol database of published proteins identified from EVs. Our result showed that 4445 proteins (86.43%) among our identified protein gene symbols were present in the database. Interestingly, 698 proteins discovered in our study were not reported in Vesiclepedia indicating that they are unique to HaCaT cells. These unique proteins may be an interesting topic of further study.

GO analysis was performed for the classification of EV-associated proteins into various molecular functions. In biological process, signal transduction and cell communication are listed as the largest classification groups. These are the basic mechanisms controlling cell growth, proliferation, and metabolism; therefore, this may indicate that EVs contribute to the proper functioning of cell migration. For instance, in one study, mitochondrial signal transduction is important in accelerating retinal wound healing [36]. In addition, the proteomic analysis strongly indicated the MAPKinase pathway as a potential target for EVs, and therefore MAPKinase activation became an interesting target of study as MAPKinase is associated with signal transduction and cell communication. Cell migration in the wound is essential for skin wound healing [4]. A previous study showed EVs from induced pluripotent stem cells promoted migration and proliferation in wound healing of diabetic mice [37]. The results in Fig. 2 show that the presence of EVs in the culture medium was associated with a more rapid decrease in the area affected by the initial wound, indicating increased migration after 48 h of exposure to EVs in both keratinocytes and fibroblasts. This result strongly implies that treatment with EVs accelerate cell proliferation and migration in vitro. The next target of this study was the mechanism behind this accelerated migration. The proteomic analysis results revealed that EVs are strongly associated with the MAPKinase pathway. Shabbir et al. demonstrated that exosomes released by human bone marrow-derived MSCs could induce the activation of several signaling pathways in target fibroblasts, including Akt and ERK1/2 [38]. A similar study from Satoh et al. reported that ERK signaling contributes to the healing of burn injuries [39]. The function of ERK1/2 and P38 is to control cell survival, migration, and apoptosis by regulating the activity of transcription factors [40]. Figure 3 shows that exposure to EVs was associated with higher level of activated ERK1/2 and P38. These results are interesting from a therapeutic point of view, as activation of ERK and P38 activity may be a promising target in wound healing in the future.

To confirm the specificity of the identified pathway, keratinocytes were pretreated with PD98059 or SB239063. The result showed the effect of EVs can be inhibited by these compounds, suggesting that EVs may affect upstream components of the MAPKinase pathway rather than ERK or P38 themselves. In addition, EVs act on multiple components of the MAPKinase pathway as treatment with both MEK1/2 and P38 inhibitors, thereby blocking the majority of MAPKinase pathway, was required to fully mitigate the accelerated migration found in EV-treated cells. In contrast, treatment with only one inhibitor still showed a significantly accelerated migration compared to the untreated control which indicates that EVs are acting through multiple routes of the MAPKinase signaling pathway. Further research is needed to find the specific molecules being acted upon by EVs.

Wound healing is a complex mechanism [31]. Wound healing, as a normal biological process in the human body, is accomplished by four precisely and highly programmed phases: hemostasis, inflammation, proliferation, and remodeling. To heal properly, all four phases must occur in the correct sequence and time period. Many factors can interfere with one or more phases of this process, resulting in improper or impaired wound healing [41]. A previous study demonstrated that exosomes secreted by macrophage cells influenced inflammatory pathways and contributed to the resolution of inflammation in the recipient cells [42]. In cell proliferation and migration, EVs as a form of cell communication may have an advantage that will contribute to wound healing. EV cargo analysis is a promising diagnostic/prognostic method and is great interest at present [43]. There is however, no “gold standard” for EV isolation, and the nomenclature of the EV remains unclear. This is a major problem facing the field, and although the implementation and evaluation of emerging technology is discussed, the findings of EV studies remain the same. However, with these findings and future research, we might be on the road to developing new methods to treat wounds and accelerate wound healing in the future. In vivo study will be needed in the future to confirm the molecular mechanism in wound healing.

## Conclusion

This study shows that keratinocyte-derived EVs accelerate migration and proliferation of in vitro cultured fibroblasts and keratinocytes. In addition, this accelerated migration is likely due to activation of multiple ERK and P38 upstream components of the MAPKinase signaling cascade. Although the specific molecules activated still remain unknown, with further research, EVs may be a promising new alternative to conventional methods of wound treatment.

## Supplementary Information

**Additional file 1: Supplementary Figure 1.** Determination of optimal EV concentration for cell migration assay.

**Additional file 2: Supplementary Figure 2.** Image data describing involvement of MAPKinase in cell migration in vitro.

**Additional file 3: Supplementary Figure 3.** MAPKinase inhibitor alone does not significantly affect cell migration.

**Additional file 4: Supplementary Table 1.** HaCaT extracellular vesicle unique proteins identified by comparison against Vesiclepedia.

## Data Availability

The datasets used and/or analyzed during the current study are available from the corresponding author on reasonable request.

## Abbreviations

EV: Extracellular vesicle
MAPKinease: Mitogen-activated protein kinase
ERK1/2: Extracellular regulate kinase1/2
HaCaT: Human keratinocytes
VEGF: Vascular endothelial growth factor
GO: Gene ontology
LC-MS/MS: Liquid chromatography with tandem mass spectrometry
CL: Cell lysate
MSC: Mesenchymal stem cells
DNA: Deoxyribonucleic acid
RNA: Ribonucleic acid
DMEM: Dulbecco’s modified Eagle’s medium
NHDF: Normal human dermal fibroblasts
FBS: Fetal bovine serum
TIM-4: Type I transmembrane protein 4
RIPA: Radioimmunoprecipitation assay
EDTA: Ethylenediaminetetraacetic acid
DIA: Data-independent acquisition
