# Supplementary material for: Human keratinocyte-derived extracellular vesicles activate the MAPKinase pathway and promote cell migration and proliferation in vitro
Source: Inflamm Regen. 2021 Feb 2;41:4. doi: 10.1186/s41232-021-00154-x (PMC7852286; doi:10.1186/s41232-021-00154-x)
Supplement: Supplementary file 1 — Additional file 1: Supplementary Figure 1. Determination of optimal EV concentration for cell migration assay. [file 41232_2021_154_MOESM1_ESM.docx]

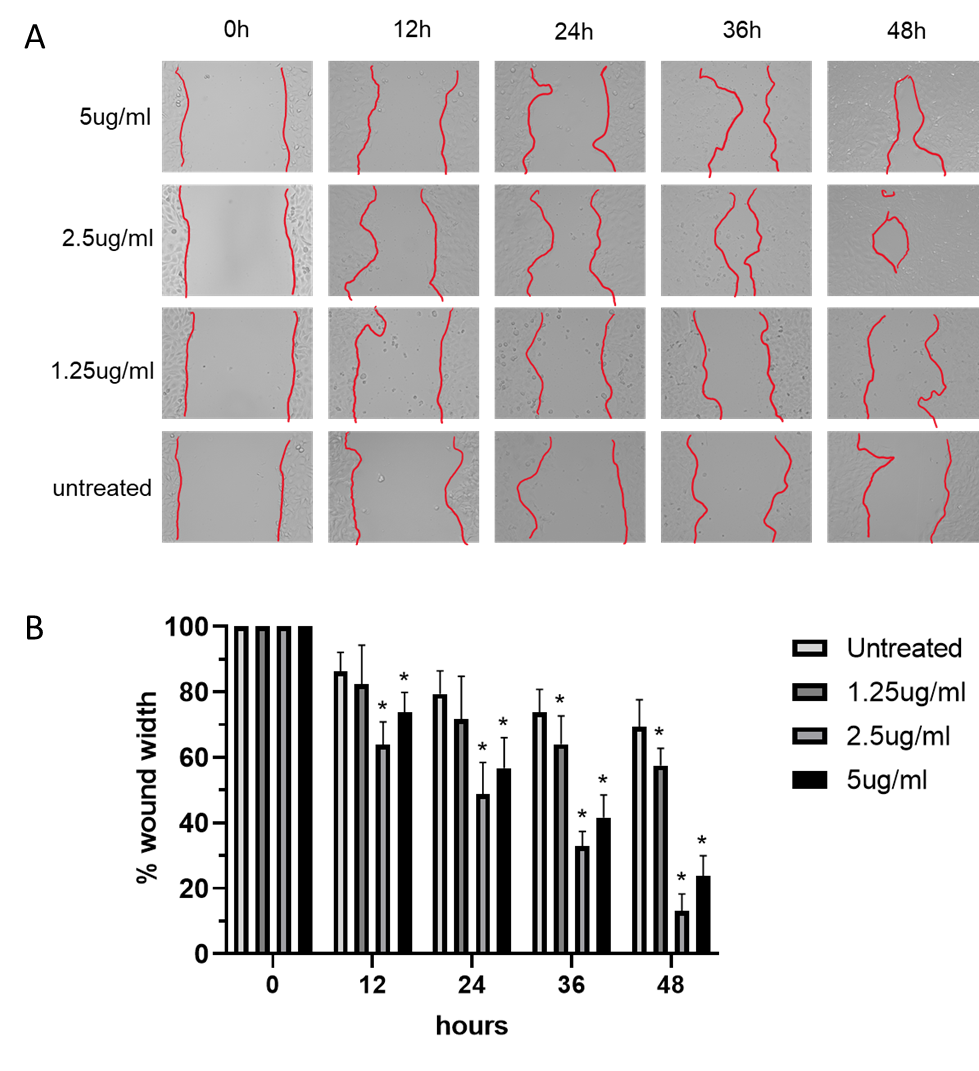


**Supplementary Figure 1: Optimization of EV concentration for migration assay**. (A) wound healing assay was performed in HaCaT cells and (B) cell migration was measured 48 hours after scratching (n = 24 scratches)
