# Supplementary material for: Human keratinocyte-derived extracellular vesicles activate the MAPKinase pathway and promote cell migration and proliferation in vitro
Source: Inflamm Regen. 2021 Feb 2;41:4. doi: 10.1186/s41232-021-00154-x (PMC7852286; doi:10.1186/s41232-021-00154-x)
Supplement: Supplementary file 2 — Additional file 2: Supplementary Figure 2. Image data describing involvement of MAPKinase in cell migration in vitro. [file 41232_2021_154_MOESM2_ESM.docx]

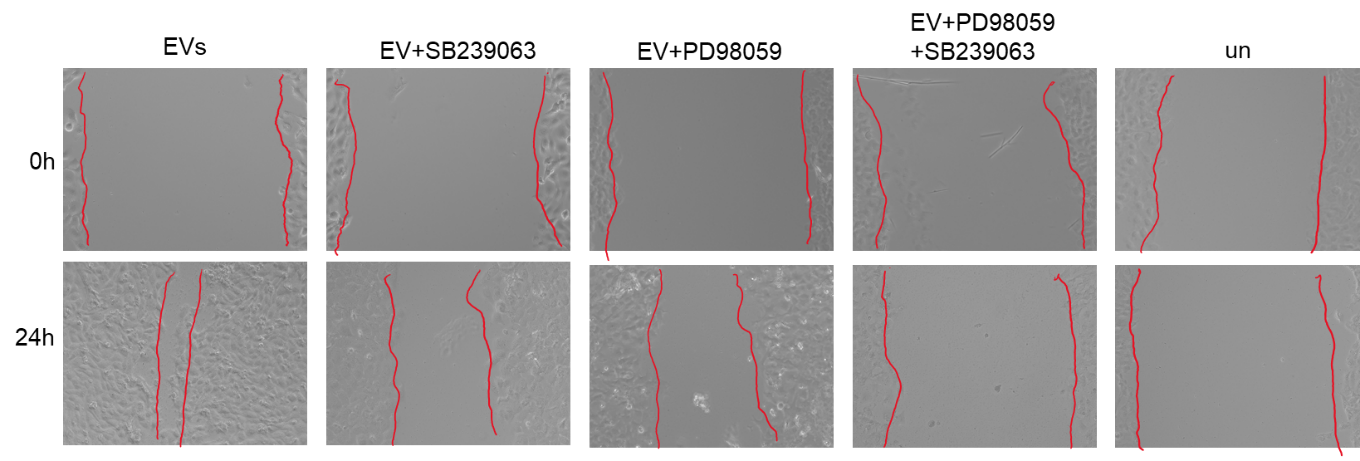


**Supplementary Figure 2: Involvement of MAPKinase in cell migration *in vitro***. Wound healing assay in HaCaT cells results after exposure to HaCaT cell derived EVs in the presence and absence of MAPKinase pathway inhibitors for 48 hours (n = 8 scratches)
