# Supplementary material for: Human keratinocyte-derived extracellular vesicles activate the MAPKinase pathway and promote cell migration and proliferation in vitro
Source: Inflamm Regen. 2021 Feb 2;41:4. doi: 10.1186/s41232-021-00154-x (PMC7852286; doi:10.1186/s41232-021-00154-x)
Supplement: Supplementary file 3 — Additional file 3: Supplementary Figure 3. MAPKinase inhibitor alone does not significantly affect cell migration. [file 41232_2021_154_MOESM3_ESM.docx]

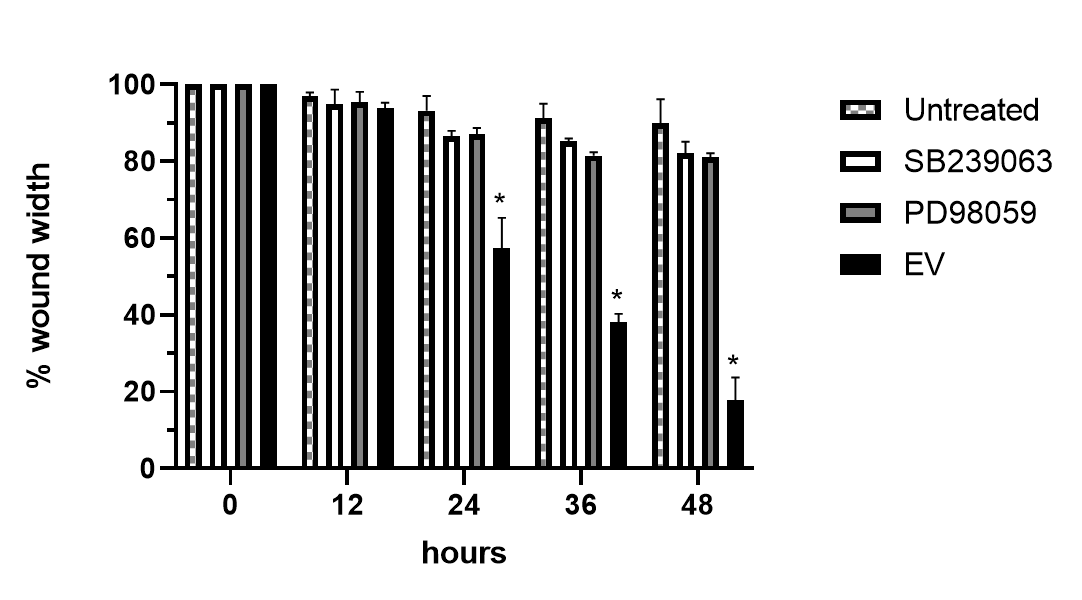


**Supplementary Figure 3: Inhibitor alone did not significantly affect cell migration**. HaCaT cell scratch assay results after exposure to MEK1/2 and P38 inhibitors without the presence of EVs for 48 hours (n = 8 scratches)
